# Association of 1-Year Blood Pressure Variability With Long-term Mortality Among Adults With Coronary Artery Disease: A Post Hoc Analysis of a Randomized Clinical Trial

**DOI:** 10.1001/jamanetworkopen.2021.8418

**Published:** 2021-04-29

**Authors:** Osama Dasa, Steven M. Smith, George Howard, Rhonda M. Cooper-DeHoff, Yan Gong, Eileen Handberg, Carl J. Pepine

**Affiliations:** 1 Department of Internal Medicine and Epidemiology, College of Public Health and Health Professions, College of Medicine, University of Florida, Gainesville; 2 Center for Integrative Cardiovascular and Metabolic Diseases, University of Florida, Gainesville; 3 Department of Pharmacotherapy and Translational Research, University of Florida College of Pharmacy, Gainesville; 4 Department of Biostatistics, University of Alabama at Birmingham, Birmingham; 5 Division of Cardiovascular Medicine, Department of Medicine, College of Medicine, University of Florida, Gainesville

## Abstract

**Importance:**

Accumulating evidence indicates that higher blood pressure (BP) variability from one physician office visit to the next (hereafter referred to as visit-to-visit BP variability) is associated with poor outcomes. Short-term measurement (throughout 1 year) of visit-to-visit BP variability in high-risk older patients may help identify patients at increased risk of death.

**Objective:**

To evaluate whether short-term visit-to-visit BP variability is associated with increased long-term mortality risk.

**Design, Setting, and Participants:**

The US cohort of the International Verapamil SR-Trandolapril Study (INVEST), a randomized clinical trial of 16 688 patients aged 50 years or older with hypertension and coronary artery disease, was conducted between September 2, 1997, and December 15, 2000, with in-trial follow-up through February 14, 2003. The study evaluated a calcium antagonist (sustained-release verapamil plus trandolapril) vs β-blocker (atenolol plus hydrochlorothiazide) treatment strategy. Blood pressure measurement visits were scheduled every 6 weeks for the first 6 months and biannually thereafter. Statistical analysis was performed from September 2, 1997, to May 1, 2014.

**Exposures:**

Visit-to-visit systolic BP (SBP) and diastolic BP variability during the first year of enrollment using 4 different BP variability measures: standard deviation, coefficient of variation, average real variability, and variability independent of the mean.

**Main Outcomes and Measures:**

All-cause death, assessed via the US National Death Index, beginning after the exposure assessment period through May 1, 2014.

**Results:**

For the present post hoc analysis, long-term mortality data were available on 16 688 patients (9001 women [54%]; mean [SD] age, 66.5 [9.9] years; 45% White patients, 16% Black patients, and 37% Hispanic patients). During a mean (SD) follow-up of 10.9 (4.2) years, 5058 patients (30%) died. All 4 variability measures for SBP were significantly associated with long-term mortality after adjustment for baseline demographic characteristics and comorbidities. After comparison of lowest vs highest variability measure quintiles, the magnitude of the association with death remained statistically significant even after adjustment for baseline demographic characteristics and comorbidities (average real variability: adjusted hazard ratio [aHR], 1.18; 95% CI, 1.08-1.30; standard deviation: aHR, 1.14; 95% CI, 1.04-1.24; coefficient of variation: aHR, 1.15; 95% CI, 1.06-1.26; variability independent of the mean: aHR, 1.15; 95% CI, 1.05-1.25). The signal was stronger in women compared with men. Associations of diastolic BP variability measures with death were weaker than for SBP and were not significant after adjustment.

**Conclusions and Relevance:**

This study suggests that, in a large population of older patients with hypertension and coronary artery disease, short-term visit-to-visit SBP variability was associated with excess long-term mortality, especially for women. Efforts to identify and minimize visit-to-visit SBP variability may be important in reducing excess mortality later in life.

**Trial Registration:**

ClinicalTrials.gov Identifier: NCT00133692

## Key Points

**Question:**

Is short-term blood pressure variability from one physician office visit to the next (hereafter referred to as visit-to-visit blood pressure variability) associated with increased long-term mortality risk?

**Findings:**

In this cohort study, 16 688 patients with hypertension who were 50 years of age or older with coronary artery disease were followed up for a mean of 10.9 years. When comparing quintiles of systolic blood pressure variability measures, higher quintiles were associated with long-term mortality even after adjusting for baseline demographic characteristics, mean blood pressure, and comorbidities; the signal was stronger in women compared with men.

**Meaning:**

These findings support efforts to identify and minimize visit-to-visit blood pressure variability to potentially reduce excess mortality later in life.

## Teaser

This cohort study evaluates whether short-term blood pressure variability from 1 physician office visit to the next is associated with increased long-term mortality risk among adults with coronary artery disease (CAD).

## Introduction

Blood pressure (BP) measured during a physician office visit (hereafter referred to as office BP) remains the usual measurement for diagnosing and monitoring hypertension worldwide. However, it is questionable whether the usual BP measurement alone can account for all future BP-related cardiovascular events.^1^ Blood pressure variability has emerged as 1 major, informative measure. Office BP is known to vary substantially from visit to visit (heareafter referred to as visit-to-visit BP variability), which is often attributed to random variation around the patient’s “true” BP owing to measurement errors.^2,3,4^ Nonetheless, accumulating evidence indicates that increased visit-to-visit BP variability is associated with worse cardiovascular disease (CVD)^5,6,7,8,9^ outcomes independent of mean BP and other CVD risk factors.

Whether short-term, visit-to-visit BP variability (calculated throughout 1 year) portends increased risk of long-term, all-cause mortality is less well established. Although hypertension is the most prevalent modifiable risk for coronary artery disease (CAD), previous BP variability studies have focused mostly on patients without CAD.^10,11,12,13,14,15,16,17,18,19^ In addition, findings from earlier studies have been mixed; some found that higher visit-to-visit BP variability was associated with increased mortality,^9,20,21^ whereas others showed no association.^1^ Also, most studies had few patients that focused on relatively short-term outcomes, populations without CAD, and relatively younger populations, and many were observational cohorts without a standardized BP measurement.^20,22^ Moreover, to our knowledge, there is a paucity of information regarding associations between short-term BP variability and long-term, all-cause mortality among older patients, particularly women, with CAD. Accordingly, we evaluated associations between short-term BP variability patterns and long-term, all-cause mortality among US patients enrolled in the International Verapamil SR-Trandolapril Study (INVEST).^23^

## Methods

### Population

The present data were obtained from a post hoc analysis of the results from INVEST. The INVEST design and primary and secondary outcomes are published in detail.^23,24^ In brief, INVEST was an international prospective, randomized, blinded end point evaluation design trial that enrolled patients between September 2, 1997, and December 15, 2000, with in-trial follow-up through February 14, 2003. Eligible patients aged 50 years or older with hypertension and stable CAD were randomly assigned to receive either a calcium antagonist–based (sustained-release verapamil plus trandolapril) or β-blocker–based (atenolol plus hydrochlorothiazide) strategy, with protocolized titration to achieve a target BP of less than 140/90 mm Hg (<130/85 mm Hg for those with kidney impairment or diabetes). Patients were assessed for treatment response, compliance, and adverse effects during scheduled protocol visits every 6 weeks for the first 6 months and biannually thereafter. The mean (SD) patient follow-up was 2.7 (1.1) years (range, 1 day-5.4 years). The BP measurement was in accordance with prior Joint National Committee on Prevention, Detection, Evaluation, and Treatment of High Blood Pressure recommendations.^25^ In brief, when performing BP measurements, a quiet room was used, with the patient performing minimal extraneous activity, sitting with uncrossed legs and feet flat on the floor. Blood pressure was measured twice using a standard mercury sphygmomanometer with the appropriate BP cuff size, separated by at least 2 minutes, and investigators were instructed to measure BP in a given patient at approximately the same time of day throughout the trial. The primary outcome was the first occurrence of death, nonfatal myocardial infarction, or nonfatal stroke. The outcomes comparing strategies were equivalent, and excellent BP control (approximately 72%) was achieved in each treatment group^23^; thus, for this analysis, patients from both treatment groups were pooled into a single cohort. The antihypertensive drugs and doses used are detailed elsewhere.^23^ INVEST was conducted according to principles of the Declaration of Helsinki.^26^ Institutional review boards and ethics committees at the participating sites^23^ approved the protocol, and patients provided written informed consent.

For this study, the outcome of interest was all-cause mortality. INVEST was planned and powered for US-only analyses, and data on long-term, all-cause death were not available for non-US participants; thus, the present study included only the US cohort. All-cause death was ascertained through the US National Death Index (NDI) search.^27^ Possible matches were identified according to NDI guidelines.^27^ To be considered a confirmed death, we required 4 or more of 5 matches among Social Security number, name, date of birth, city, and state in the NDI. Follow-up for all-cause mortality began at each patient’s 1-year visit and ended on the date of death or date of the NDI search (2014). Patients who died before the 1-year visit were excluded from all analyses (eFigure 1 in the Supplement). This analysis followed the Strengthening the Reporting of Observational Studies in Epidemiology (STROBE) reporting guideline.

### Main Exposure: BP Variability

Using all available BP data from protocol-specified visits in the first year of the trial, we investigated 4 visit-to-visit BP variability measures: (1) standard deviation (SD); (2) coefficient of variation (CV), which is the SD divided by the mean; (3) average real variability (ARV) across multiple visits assessed by calculating the mean absolute differences between successive BP measurements in each clinic visit, taking into account the order of BP measurements (in contrast to CV and SD) and tending to be less affected by trends^28^; and (4) variability independent of the mean (VIM). The latter was computed by dividing an individual’s SD to the mean^x^ and multiplying it by the population’s mean^x^, where “x” is derived by fitting a regression curve through a plot of SD against the mean (eBox in the Supplement).^29^

The SD, CV, and ARV are necessarily correlated with mean BP.^30^ In addition, SD and CV are associated with visit-to-visit BP outlier values, whereas ARV accounts for between-visit time intervals and BP measurement order. VIM is constructed to have no correlation with the mean BP but is dependent on the distribution of the BP within a cohort.^7^ Prior evidence suggests that SD, CV, and VIM are highly correlated and may convey similar information about variability around the mean BP across visits. Conversely, because ARV captures variability from one visit to another, it may convey different information.^30^ To assess these correlations in our high-risk, sex-diverse, and racially/ethnically diverse older cohort, we calculated all 4 metrics, capturing variability between consecutive visits and overall variability.

### Statistical Analysis

Statistical analysis was performed from September 2, 1997, to May 1, 2014. Categorical variables were presented as frequencies and percentages, and continuous variables were presented as mean (SD) values. We aimed to investigate the association between each short-term, visit-to-visit BP variability measure and long-term mortality. We also estimated the association between mean BP (during the same time frame) and long-term mortality.

To calculate BP variability traits for each participant, we used systolic BP (SBP) and diastolic BP (DBP) measurements from clinic visit number 1 (baseline) through visit number 6 (1 year after randomization) (eFigure 1 in the Supplement). We first calculated SD, CV, and ARV. To calculate VIM, we derived the power “x” using the PROC NLIN procedure in SAS, version 9.4 (SAS Institute Inc), and then we used the formula to compute VIM for each patient. Pearson correlation coefficients were used to evaluate associations between different BP variability measures and mean BP.

For each variability measure, we categorized patients into quintiles to compare different degrees of BP variability in association with mortality using the lowest quintile as a reference. The Kaplan-Meier method assessed the cumulative incidence of death for each variability measure. We used Cox proportional hazards regression analysis to calculate crude and adjusted hazard ratios (aHRs) and 95% CIs, with long-term mortality as the dependent variable and each BP variability measure as the primary independent variable of interest, in separate models. We also assessed similar prediction models with the mean BP (during 1 year) as the predictor. Covariates included in all adjusted models were age, sex, race/ethnicity, study site, treatment assignment group, body mass index, mean BP, history of diabetes, hypercholesterolemia, prior myocardial infarction, coronary artery bypass graft surgery or percutaneous coronary revascularization, heart failure, left ventricular hypertrophy, peripheral arterial disease, prior transient ischemic attack or stroke, and kidney insufficiency. These covariates were selected a priori as evidence demonstrated that they are associated with hypertension and mortality.^31,32^ We tested for heterogeneity in the association between each BP variability trait and the primary outcome by sex, race/ethnicity, and treatment assignment group by including multiplicative interaction terms. Stratified analyses were considered when a statistically significant interaction was present (*P* < .05). We used the Cochran-Armitage test for trend to evaluate dose-response association across quintiles of BP variability measures with mortality, even if the association was not statistically significant for any particular exposure level. We also modeled the association of BP variability measures as a continuous variable, with long-term mortality evaluated using Cox proportional hazards regression models and restricted quadratic splines with knots at the 5th, 27.5th, 50th, 72.5th, and 95th percentiles of each BP variability measure. All analyses were performed using SAS, version 9.4. A 2-sided *P* < .05 was considered statistically significant.

## Results

Of 22 576 patients enrolled in INVEST between September 2, 1997, and December 15, 2000,^23^ 17 131 resided in the US. The 16 688 patients alive at the end of the first year of follow-up contributed to this analysis. Their mean (SD) age was 66.5 (9.9) years, and 54% were women. The population was racially/ethnically diverse, with 45% White participants, 37% Hispanic participants, and 16% Black participants. The mean (SD) body mass index (calculated as weight in kilograms divided by height in meters squared) was 29.5 (5.8); 29% of participants had a history of diabetes, 29% had prior myocardial infarction, and 29% had undergone revascularization (Table 1). In-trial follow-up continued through 2003, with extended follow-up for mortality through 2014. Pertinent baseline characteristics of patients in the original INVEST cohort are presented in eTable 1 in the Supplement.

**Table 1.** Pertinent Baseline Patient Characteristics

Characteristic	Patients, No. (%) (N = 16 688)
Age, mean (SD), y	66.5 (9.9)
BMI, mean (SD)	29.5 (5.8)
Sex	
Female	9001 (54)
Male	7687 (46)
Race/ethnicity	
White	7518 (45)
Black	2587 (16)
Hispanic	6109 (37)
Other^a^	474 (3)
Baseline, mean (SD), mm Hg	
SBP	148.2 (19.0)
DBP	85.0 (11.0)
Overall mean (SD), mm Hg	
SBP	139.7 (13.7)
DBP	80.4 (7.9)
History of	
Diabetes^b^	4896 (29)
Hypercholesterolemia^b^	9337 (56)
Renal insufficiency^c^	326 (2)
Prior MI^d^	4814 (29)
Congestive heart failure	832 (5)
Coronary revascularization (CABG and/or PCI)	4885 (29)
Evidence of LVH	2632 (16)
Peripheral artery disease	2145 (13)
TIA or stroke	1238 (7)

Abbreviations. BMI, body mass index (calculated as weight in kilograms divided by height in meters squared); CABG, coronary artery bypass graft surgery; DBP, diastolic blood pressure; LVH, left ventricular hypertrophy; MI, myocardial infarction; PCI, percutaneous coronary intervention; SBP, systolic blood pressure; TIA, transient ischemic attack.

^a^ Asian and other or multiracial.

^b^ History of or currently taking antidiabetic or lipid-lowering medication.

^c^ History of or currently have elevated serum creatinine level but less than 4 mg/dL (to convert to micromoles per liter, multiply by 88.4).

^d^ Remote confirmed MI (≥3 months prior to enrollment).

The mean (SD) number of BP measurement visits was 4.0 (1.6), and the mean (SD) follow-up duration was 255.9 (90.2) days. The mean baseline SBP (SD) was 148.2 (19.0) mm Hg, and the mean (SD) baseline DBP was 85.0 (11.0) mm Hg (Table 1). During 1 year of follow-up, the overall mean (SD) SBP was 139.7 (13.7) mm Hg, and the overall mean (SD) DBP was 80.4 (7.9) mm Hg. Correlations among BP variability measures are summarized in eTables 2 and 3 in the Supplement. For SBP, VIM was highly correlated with SD and ARV (Pearson *r* = 0.95 and 0.77, respectively) but not mean SBP (Pearson *r* = 0.016). Qualitatively similar findings were observed for DBP variability measures.

**Figure 1.**
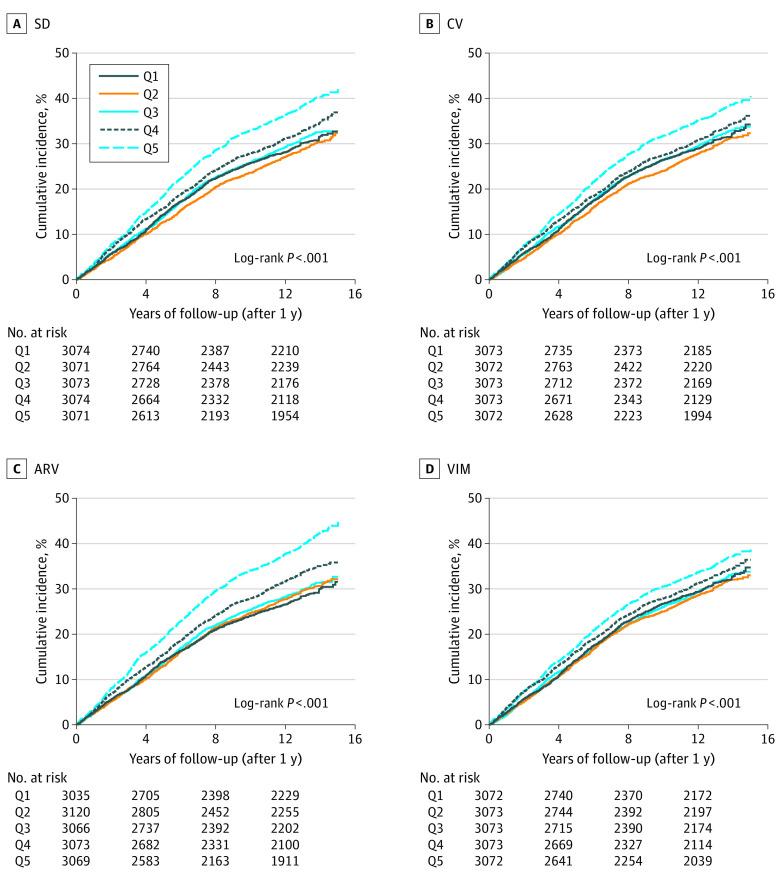
Kaplan-Meier Plots for Systolic Blood Pressure (SBP) Variability and Long-term Mortality Kaplan-Meier cumulative hazard curves for long-term, all-cause mortality outcome as a function of 4 different SBP variability measures, each divided into quintiles. ARV indicates average real variability; CV, coefficient of variation; Q1, lowest (reference) quintile; Q5, highest quintile; SD, standard deviation; and VIM, variability independent of the mean.

During a mean (SD) follow-up of 10.9 (4.2) years, 5058 deaths occurred (27.9 deaths per 1000 person-years). Mean BP was weakly associated with mortality for SBP measurements (aHR per 1 mm Hg, 1.007; 95% CI, 1.005-1.009) and DBP measurements (aHR, 1.005; 95% CI, 1.001-1.009). In general, the cumulative incidence of all-cause mortality was progressively higher at higher levels of SBP variability for all measures (Figure 1). In unadjusted Cox proportional hazards regression models, the HRs for long-term mortality increased, in a gradated fashion, across quintiles of all 4 SBP variability measures (Table 2). After adjustment for baseline demographic characteristics and comorbidities, the HRs for SBP variability were somewhat diminished but remained significant when comparing the highest quintile with the lowest quintile for all 4 variability measures (ARV: aHR, 1.18; 95% CI, 1.08-1.30; SD: aHR, 1.14; 95% CI, 1.04-1.24; CV: aHR, 1.15; 95% CI, 1.06-1.26; VIM: aHR, 1.15; 95% CI, 1.05-1.25). ARV had the strongest association with mortality in adjusted analyses among all variability measures (Table 2).

**Table 2.** All-Cause Mortality Associated With Systolic Blood Pressure Variability Measures^a^

Quintile	Hazard ratio (95% CI)							
	Variability independent of the mean		Average real variability		Coefficient of variation		Standard deviation	
	Crude	Adjusted	Crude	Adjusted	Crude	Adjusted	Crude	Adjusted
1	1 [Reference]	1 [Reference]	1 [Reference]	1 [Reference]	1 [Reference]	1 [Reference]	1 [Reference]	1 [Reference]
2	0.97 (0.88-1.06)	0.97 (0.88-1.06)	1.04 (0.95-1.14)	0.99 (0.90-1.08)	0.95 (0.87-1.04)	0.95 (0.87-1.04)	0.95 (0.87-1.04)	0.93 (0.85-1.02)
3	1.01 (0.92-1.10)	0.96 (0.88-1.05)	1.06 (0.97-1.16)	0.95 (0.87-1.04)	1.03 (0.94-1.12)	0.98 (0.90-1.07)	1.04 (0.95-1.14)	0.97 (0.89-1.06)
4	1.08 (0.99-1.18)	1.04 (0.96-1.14)	1.22 (1.12-1.34)	1.05 (0.95-1.14)	1.08 (0.99-1.18)	1.02 (0.93-1.11)	1.13 (1.03-1.23)	1.02 (0.94-1.12)
5	1.18 (1.08-1.29)	1.15 (1.05-1.25)	1.53 (1.40-1.67)	1.18 (1.08-1.30)	1.26 (1.15-1.37)	1.15 (1.06-1.26)	1.36 (1.25-1.48)	1.14 (1.04-1.24)

^a^ Results of Cox proportional hazards regression analysis (hazard ratios and 95% CIs) for all-cause mortality comparing higher systolic blood pressure variability measures in quintiles, with the lowest quintile as a reference. Higher quintiles were associated with higher mortality. This association continued to be significant after adjustment. Covariates included in all adjusted models were age, sex, race/ethnicity, study site, treatment assignment group, body mass index, mean blood pressure, history of diabetes, hypercholesterolemia, history of prior myocardial infarction, prior coronary artery bypass graft surgery or percutaneous coronary revascularization, heart failure, left ventricular hypertrophy, peripheral arterial disease, prior transient ischemic attack or stroke, and history of renal insufficiency.

Interaction was significant between VIM and sex, and in analyses stratified by sex, women exhibited stronger associations between BP variability and the primary outcome than men (eTables 4 and 5 in the Supplement). There was no significant interaction between each BP trait and race/ethnicity nor treatment assignment group. However, owing to small *P* values for interaction, we also stratified the analysis by treatment groups, which revealed a predilection for stronger association with the primary outcome in the calcium antagonist–based group vs the β-blocker group (eTables 6 and 7 in the Supplement, respectively). There were trivial differences in baseline characteristics across quintiles of SBP variability measures (eTables 8-11 in the Supplement). Mortality progressively increased when SBP variability measures were modeled as continuous variables (eFigure 2 in the Supplement). A *P* value for trend was statistically significant for all SBP variability measures (eTable 12 in the Supplement).

**Figure 2.**
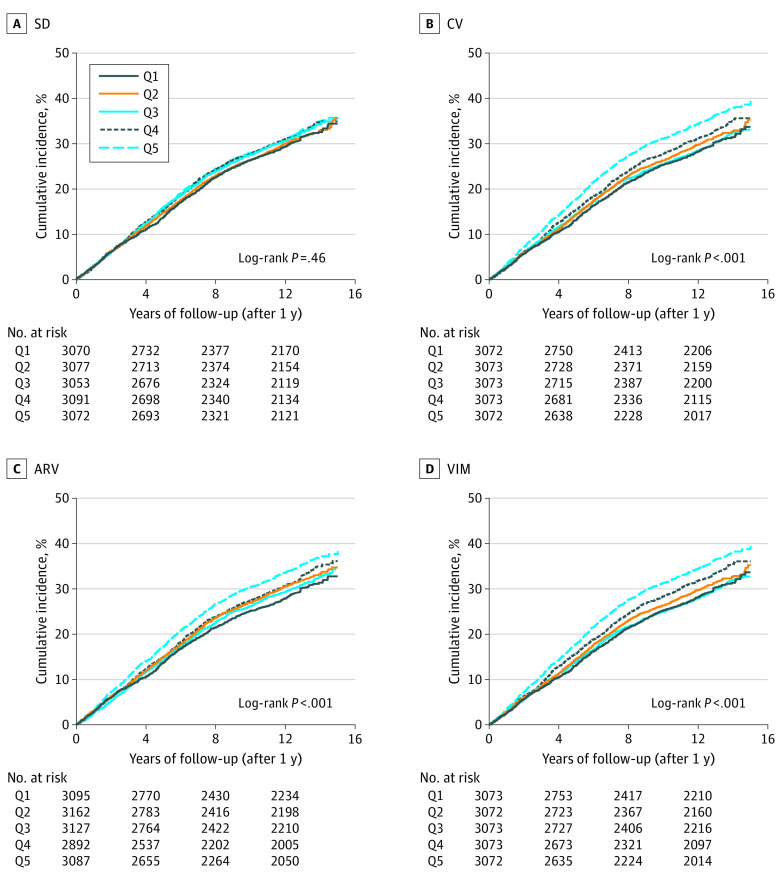
Kaplan-Meier Curves for Diastolic Blood Pressure (DBP) Variability and Long-term Mortality Kaplan-Meier cumulative hazard curves for long-term mortality outcomes as a function of 4 different DBP variability measures, each divided into quintiles. ARV indicates average real variability; CV, coefficient of variation; Q1, lowest (reference) quintile; Q5, highest quintile; SD, standard deviation; and VIM, variability independent of the mean.

In analyses of DBP variability, only VIM, ARV, and CV showed a gradated response between increasing variability and increasing risk of death (Figure 2). Moreover, crude HRs for DBP variability across all variability measures were lower than those observed for SBP (Table 3). After adjustment, DBP variability was not associated with mortality for any of the variability measures. eFigure 3 in the Supplement displays DBP variability measures modeled as continuous variables.

**Table 3.** All-Cause Mortality Associated With Diastolic Blood Pressure Variability Measures^a^

Quintile	Hazard ratio (95% CI)							
	Variability independent of the mean		Average real variability		Coefficient of variation		Standard deviation	
	Crude	Adjusted	Crude	Adjusted	Crude	Adjusted	Crude	Adjusted
1	1 [Reference]	1 [Reference]	1 [Reference]	1 [Reference]	1 [Reference]	1 [Reference]	1 [Reference]	1 [Reference]
2	1.05 (0.96-1.15)	0.95 (0.87-1.04)	1.08 (0.99-1.18)	0.95 (0.87-1.04)	1.05 (0.96-1.15)	0.95 (0.87-1.03)	1.01 (0.93-1.10)	0.93 (0.85-1.02)
3	1.00 (0.91-1.09)	0.91 (0.83-1.00)	1.04 (0.95-1.14)	0.92 (0.84-1.00)	1.01 (0.93-1.11)	0.94 (0.85-1.02)	1.05 (0.96-1.15)	0.98 (0.90-1.07)
4	1.14 (1.05-1.25)	1.02 (0.93-1.11)	1.12 (1.02-1.23)	0.99 (0.91-1.08)	1.12 (1.02-1.22)	1.00 (0.91-1.09)	1.06 (0.97-1.16)	1.01 (0.93-1.11)
5	1.27 (1.16-1.38)	1.07 (0.98-1.17)	1.23 (1.13-1.34)	1.07 (0.98-1.17)	1.26 (1.16-1.37)	1.08 (0.99-1.17)	1.07 (0.98-1.17)	1.04 (0.96-1.14)

^a^ Results of Cox proportional hazards regression analysis (hazard ratios and 95% CIs) for all-cause mortality comparing higher diastolic blood pressure variability measures in quintiles, with the lowest quintile as a reference. Higher quintiles were associated with higher mortality, but this association became attenuated and insignificant after adjustment. Covariates included in all adjusted models were age, sex, race/ethnicity, study site, treatment assignment group, body mass index, mean blood pressure, history of diabetes, hypercholesterolemia, history of prior myocardial infarction, prior coronary arterty bypass graft surgery or percutaneous coronary revascularization, heart failure, left ventricular hypertrophy, peripheral arterial disease, prior transient ischemic attack or stroke, and history of renal insufficiency.

## Discussion

INVEST was the largest randomized clinical trial of older patients with hypertension (approximately one-third >70 years^33^) with CAD. In this high-risk population, short-term (within the first year after randomization), visit-to-visit SBP variability, but not DBP variability, was associated with long-term mortality. Our findings remained consistent even after adjustment for a robust set of well-measured potential confounders. Compared with the lowest quintiles, the highest quintiles of SBP variability measures were associated with a 14% to 18% increased risk of long-term mortality. Across all 4 BP variability measurements, the signal was significantly stronger among women, a cohort often underenrolled in CVD trials. Our data add to and expand the growing evidence of the prognostic value of visit-to-visit BP variability and its association with poor outcomes for older patients with hypertension and CAD.

Until the late 1990s, variability in BP was considered a random phenomenon that needed to be overcome to determine the “true BP,” especially in clinical trials.^2^ INVEST used a computer-based algorithm with an important “validation logic” built in to identify and exclude nonphysiological or highly variable BP responses.^23^ Despite the exclusion of extreme BP variability, we observed that visit-to-visit SBP variability was associated with long-term mortality up to almost 13 years after the assessment of SBP variability. These data are consistent with reports that visit-to-visit variability in BP is not a merely random phenomenon and has important prognostic implications in some other cohorts.^34,35^

Prior studies document an association of higher BP variability with CVD,^7,8,9^ stroke,^5,6,7^ left ventricular remodeling,^36^ and death.^21,28,37,38,39,40,41^ However, most of these studies have focused on relatively short-term outcomes, populations without a prior history of CAD, and relatively younger populations.^20,22^ Those studies also had relatively fewer patients and shorter follow-up durations, and many were observational cohorts without a standardized BP measurement. Also, owing to wide heterogeneity of prior studies, the number of BP visits, the time between visits, and the cohort size, conflicting results emerged regarding the associations between BP variability and adverse outcomes.^21,22,42,43,44,45^ Our study attempted to address some of the limitations of the prior studies by evaluating the long-term mortality of a large, older, high-risk population (16 688 patients; mean [SD] age, 66.5 [9.9] years) with a similar representation of men and women. Our population was well characterized, with protocolized BP measurements. Furthermore, the mortality signal with SBP variability was consistent even after adjustment for multiple baseline covariates. This association, as in prior BP variability studies,^7^ was probably not associated with poor BP control because approximately 72% of INVEST patients achieved goal BP levels by the end of trial follow-up.^23^

Many theories have been proposed to explain the underlying pathologic mechanisms associated with higher BP variability. Animal models suggest greater arterial remodeling, endothelial dysfunction, vascular injury, and coronary atheroma progression.^46,47^ Others have suggested that β_2_-adrenoceptor desensitization is associated with less β_2_-mediated arterial relaxation.^48^ Moreover, greater BP variability was associated with myocardial fibrosis, hypertrophy, and dysfunction in humans and animal models.^36,49^ All of these factors may play a role in the long-term increase in mortality observed with higher BP variability.

Prior studies reported that female sex, older age, obesity, “white coat hypertension” (elevated office or clinic BP levels induced by the presence of the physician or health care professional), and certain antihypertensive medications are associated with higher BP variability patterns.^21,22,50^ In addition, mechanisms have been proposed to explain higher visit-to-visit BP variability, including impaired baroreceptor function,^51^ inflammation,^52,53,54^ and kidney impairment.^55^ Higher BP variability was also bidirectionally associated with greater arterial stiffness and remodeling in human studies and population-based cohorts.^56,57,58,59^ Evidence suggests that increased arterial stiffness is associated with higher BP variability, whereas, on the other hand, increased BP variability is associated with increased arterial wall stiffness.^59,60^ Also, for women, estrogen has an intricate role to play in vascular function.^61^ It is possible that the present cohort, being older and with a higher proportion of women in menopause, had higher BP variability and possibly a stronger association with mortality. Finally, low medication adherence has been proposed as a cause of BP variability.^62^ However, adherence rates in INVEST were estimated to be 78% to 82% at 24 months and likely even higher at 12 months (the BP measurement duration in this study).^23^ Thus, we believe that it is unlikely that nonadherence played a major role.

Although BP variability is known to portend worse outcomes, it is rarely used to guide clinical practice, in part because general agreement is lacking on a reference standard to measure outpatient visit-to-visit BP variability. Some suggest that at least 6 clinic visits are needed to calculate BP variability,^63^ although the true number remains unknown and difficult to estimate.^64^ In this study, the easy-to-calculate BP variability measures were associated with mortality and are highly feasible for clinical practice through automated calculations within electronic records. Future BP variability research should focus on causal pathways of worse outcomes and interventions to reduce individual BP variability between visits. In the meantime, current electronic health systems should routinely incorporate BP variability measures in the clinic to identify patients at higher risk for future CVD events and mortality who may warrant more intensive preventive measures to mitigate their residual risk and reduce their morbidity and mortality (eFigure 4 in the Supplement).

### Strengths and Limitations

This study has several strengths. It was conducted in a large, relatively older, racially/ethnically diverse population at high risk of CAD with a similar representation of women and men. It used high-quality data with detailed phenotyping from a randomized clinical trial. Moreover, INVEST had high retention rates, detailed inclusion and exclusion criteria, a standardized BP measurement protocol, and all electronic data collection with rigorous quality control. The outcome of interest (all-cause death) is resistant to bias, clinically relevant, and patient centered. Besides the mean BP, we used 4 different BP variability measures and adjusted for a wide range of confounders in the final analysis. Last, our study had among the longest follow-up durations for the outcome of interest.

This study also has several limitations. The generalizability of the findings to other populations (those without CAD or of younger age) or to patients in real-life settings is unclear. Also unclear is the optimal number of visits and the time interval between clinic visits for BP variability assessments. We could not account for BP variability between clinic visits, out-of-office or home BP measurement, or compare manual vs automated BP measurements or the ideal number of BP measurements at each visit. Data on compliance with antihypertensive drugs during follow-up were not available. Our outcome of interest was based on death certificates, not on primary trial follow-up data, which can predispose to misclassification. Nevertheless, the NDI has been validated as an accurate measure of death in the US, particularly for those that have not occurred recently.^65^ Finally, the present data are a post hoc analysis of a randomized clinical trial, a limitation that requires caution when interpreting conclusions.

## Conclusions

Visit-to-visit BP variability remains underused in clinical settings. Although antihypertensive medications reduce SBP and DBP, higher short-term BP variability is associated with residual risk for adverse long-term mortality even among patients with controlled BP. Short-term (approximately 1 year) SBP variability measures were associated with mortality more than a decade after measurements in older patients with CAD, even when using variability measures not correlated with mean BP. This association was stronger for women. Next steps should embrace incorporating variability measures in the clinic. Further research should address this excess risk measure with novel therapies or combinations of medications to reduce mortality risk.

## Notes

Supplement. **eTable 1.** Original INVEST Study Baseline Characteristics **eTable 2.** Pearson’s Correlation Coefficients for SBP Variability Measure **eTable 3.** Pearson’s Correlation Coefficients for DBP Variability Measures **eTable 4.** Hazard Ratios and 95% Confidence Intervals for All-Cause Mortality in Males **eTable 5.** Hazard Ratios and 95% Confidence Intervals for All-Cause Mortality in Females **eTable 6.** Hazard Ratios and 95% Confidence Intervals for All-Cause Mortality in the Calcium Channel Blocker Arm **eTable 7.** Hazard Ratios and 95% Confidence Intervals for All-Cause Mortality in the Beta-Blocker Arm **eTable 8.** Pertinent Baseline Patient Characteristics Across Quintiles of VIM **eTable 9.** Pertinent Baseline Patient Characteristics Across Quintiles of ARV **eTable 10.** Pertinent Baseline Patient Characteristics Across Quintiles of CV **eTable 11.** Pertinent Baseline Patient Characteristics Across Quintiles of SD **eTable 12.** Cochran-Armitage Test for Trend Across Blood Pressure Variability Quintiles **eBox.** Blood Pressure Variability Equations **eFigure 1.** Study Timeline **eFigure 2.** Spline Curves of Systolic Blood Pressure Variability Measures on Primary Outcome **eFigure 3.** Spline Curves of Diastolic Blood Pressure Variability Measures on Primary Outcome **eFigure 4.** Schematic Diagram of Implementing BP Variability Calculation in Clinical Practice
