# Supplementary material for: Association of 1-Year Blood Pressure Variability With Long-term Mortality Among Adults With Coronary Artery Disease: A Post Hoc Analysis of a Randomized Clinical Trial
Source: JAMA Netw Open. 2021 Apr 29;4(4):e218418. doi: 10.1001/jamanetworkopen.2021.8418 (PMC8085725; doi:10.1001/jamanetworkopen.2021.8418)
Supplement: Supplement. — eTable 1. Original INVEST Study Baseline Characteristics eTable 2. Pearson’s Correlation Coefficients for SBP Variability Measure eTable 3. Pearson’s Correlation Coefficients for DBP Variability Measures eTable 4. Hazard Ratios and 95% Confidence Intervals for All-Cause Mortality in Males eTable 5. Hazard Ratios and 95% Confidence Intervals for All-Cause Mortality in Females eTable 6. Hazard Ratios and 95% Confidence Intervals for All-Cause Mortality in the Calcium Channel Blocker Arm eTable 7. Hazard Ratios and 95% Confidence Intervals for All-Cause Mortality in the Beta-Blocker Arm eTable 8. Pertinent Baseline Patient Characteristics Across Quintiles of VIM eTable 9. Pertinent Baseline Patient Characteristics Across Quintiles of ARV eTable 10. Pertinent Baseline Patient Characteristics Across Quintiles of CV eTable 11. Pertinent Baseline Patient Characteristics Across Quintiles of SD eTable 12. Cochran-Armitage Test for Trend Across Blood Pressure Variability Quintiles eBox. Blood Pressure Variability Equations eFigure 1. Study Timeline eFigure 2. Spline Curves of Systolic Blood Pressure Variability Measures on Primary Outcome eFigure 3. Spline Curves of Diastolic Blood Pressure Variability Measures on Primary Outcome eFigure 4. Schematic Diagram of Implementing BP Variability Calculation in Clinical Practice [file jamanetwopen-e218418-s001.pdf]

Supplementary Online Content

Dasa O, Smith SM, Howard G, et al. Association of 1-year blood pressure variability with long-term mortality among adults with coronary artery disease: a post hoc analysis of a randomized clinical trial. *JAMA Netw Open.* 2021;4(4):e218418. doi:10.1001/jamanetworkopen.2021.8418

eTable 1. Original INVEST Study Baseline Characteristics
eTable 2. Pearson's Correlation Coefficients for SBP Variability Measure
eTable 3. Pearson's Correlation Coefficients for DBP Variability Measures
eTable 4. Hazard Ratios and 95% Confidence Intervals for All-Cause Mortality in Males
eTable 5. Hazard Ratios and 95% Confidence Intervals for All-Cause Mortality in Females
eTable 6. Hazard Ratios and 95% Confidence Intervals for All-Cause Mortality in the Calcium Channel Blocker Arm
eTable 7. Hazard Ratios and 95% Confidence Intervals for All-Cause Mortality in the Beta-Blocker Arm
eTable 8. Pertinent Baseline Patient Characteristics Across Quintiles of VIM
eTable 9. Pertinent Baseline Patient Characteristics Across Quintiles of ARV
eTable 10. Pertinent Baseline Patient Characteristics Across Quintiles of CV
eTable 11. Pertinent Baseline Patient Characteristics Across Quintiles of SD
eTable 12. Cochran-Armitage Test for Trend Across Blood Pressure Variability Quintiles
eBox. Blood Pressure Variability Equations
eFigure 1. Study Timeline
eFigure 2. Spline Curves of Systolic Blood Pressure Variability Measures on Primary Outcome
eFigure 3. Spline Curves of Diastolic Blood Pressure Variability Measures on Primary Outcome
eFigure 4. Schematic Diagram of Implementing BP Variability Calculation in Clinical Practice

This supplementary material has been provided by the authors to give readers additional information about their work.

© 2021 Dasa O et al. *JAMA Network Open.*

eTable 1. Original INVEST Study Baseline Characteristics

Characteristic*	Calcium Antagonist Strategy (n=11,267)	Non-Calcium Antagonist Strategy (n=11,309)
**Age, years**	66.0 (9.7)	66.1 (9.8)
**Female sex**	5850 (51.9)	5920 (52.3)
**BMI, Kg/m^2**	29.1 (6.8)	29.2 (7.4)
**Race/Ethnicity**		
White	5466 (48.5)	5459 (48.3)
Black	1506 (13.4)	1523 (13.5)
Hispanic	4021 (35.7)	4024 (35.6)
Asian	63 (0.6)	86 (0.8)
Other	211 (1.9)	217 (1.9)
**History of:**		
Myocardial infarction†	3622 (32.1)	3596 (31.8)
Heart failure	619 (5.5)	637 (5.6)
Coronary Revascularization (CABG and/or PCI)	3079 (27.3)	3087(27.3)
Left ventricular hypertrophy	2422 (21.5)	2526 (22.3)
Stroke or TIA	595 (5.3)	567 (5.0)
Peripheral vascular disease	1345 (11.9)	1354 (12.0)
Diabetes mellitus‡	3169 (28.1)	3231 (28.6)
Hypercholesterolemia‡	6300 (55.9)	6293 (55.6)
Renal insufficiency§	214 (1.9)	210 (1.9)
**Past smoker**	5247 (46.6)	5207 (46.0)
**Current smoker**	1435 (12.7)	1374 (12.0)

eTable 1 was adopted from the original INVEST trial publication (JAMA. 2003;290(21):2805-2816).
*Data are expressed as mean ± SD or n (%) unless otherwise indicated.
†Remote confirmed MI (≥3 months prior to enrollment).
‡History of or currently taking antidiabetic or lipid-lowering medication.
§History of or currently have elevated serum creatinine level but less than 4 mg/dl.
Abbreviations. BMI, body mass index; n, number; CABG, coronary artery bypass surgery; INVEST, the international verapamil-trandolapril study; SD, standard deviation; TIA, trainset ischemic attack.

© 2021 Dasa O et al. *JAMA Network Open.*

eTable 2. Pearson's Correlation Coefficients for SBP Variability Measure

	MBP	SD	CV	VIM	ARV
**MBP**	1.00	0.289 (<.0001)	0.112 (<.0001)	0.016 (0.0229)	0.314 (<.0001)
**SD**	0.289 (<.0001)	1.00	0.979 (<.0001)	0.951 (<.0001)	0.846 (<.0001)
**CV**	0.112 (<.0001)	0.979 (<.0001)	1.00	0.994 (<.0001)	0.808 (<.0001)
**VIM**	0.016 (0.0229)	0.951 (<.0001)	0.994 (<.0001)	1.00	0.775 (<.0001)
**ARV**	0.313 (<.0001)	0.846 (<.0001)	0.808 (<.0001)	0.775 (<.0001)	1.00

Correlation matrix for SBP variability measures. Numbers are expressed as "r" (P-value).
Abbreviations. ARV, average real variability; CV, coefficient of variation; SBP, systolic blood pressure; MBP, mean blood pressure; SD, standard deviation; VIM, variability independent of the mean.

© 2021 Dasa O et al. *JAMA Network Open.*

eTable 3. Pearson's Correlation Coefficients for DBP Variability Measures

	MBP	SD	CV	VIM	ARV
**MBP**	1.00	0.200 (<.0001)	0.001 (0.8617)	-0.021 (0.002)	0.187 (<.0001)
**SD**	0.200 (<.0001)	1.00 (<.0001)	0.974 (<.0001)	0.968 (<.0001)	0.845 (<.0001)
**CV**	0.001 (0.862)	0.974 (<.0001)	1.00	0.999 (<.0001)	0.816 (<.0001)
**VIM**	-0.021 (0.0020)	0.968 (<.0001)	0.999 (<.0001)	1.00	0.810 (<.0001)
**ARV**	0.187 (<.0001)	0.845 (<.0001)	0.816 (<.0001)	0.810 (<.0001)	1.00

Correlation matrix for DBP variability measures. Numbers are expressed as "r" (P-value).
Abbreviations. ARV, average real variability; CV, coefficient of variation; DBP, diastolic blood pressure; MBP, mean blood pressure; SD, standard deviation; VIM, variability independent of the mean.

© 2021 Dasa O et al. *JAMA Network Open.*

eTable 4. Hazard Ratios and 95% Confidence Intervals for All-Cause Mortality in Males

	**VIM**		**ARV**		**CV**		**SD**	
Quintile	Crude	Adjusted	Crude	Adjusted	Crude	Adjusted	Crude	Adjusted
Q1	1.0 (ref)	1.0 (ref)	1.0 (ref)	1.0 (ref)	1.0 (ref)	1.0 (ref)	1.0 (ref)	1.0 (ref)
Q2	0.910 (0.81-1.03)	0.918 (0.81-1.04)	1.004 (0.89-1.14)	0.994 (0.88-1.13)	0.914 (0.81-1.03)	0.916 (0.81-1.04)	0.958 (0.85-1.08)	0.958 (0.85-1.08)
Q3	0.994 (0.88-1.12)	0.969 (0.86-1.09)	0.988 (0.87-1.12)	0.933 (0.82-1.06)	1.022 (0.91-1.15)	0.992 (0.88-1.12)	1.012 (0.90-1.14)	0.977 (0.87-1.10)
Q4	1.079 (0.96-1.22)	1.041 (0.92-1.18)	**1.379 (1.22-1.56)**	**1.117 (0.99-1.26)**	1.051 (0.93-1.19)	1.023 (0.91-1.16)	1.094 (0.97-1.24)	1.059 (0.938-1.20)
Q5	1.090 (0.96-1.23)	1.036 (0.92-1.17)	**1.379 (1.22-1.56)**	**1.174 (1.04-1.33)**	**1.190 (1.06-1.34)**	1.096 (0.97-1.24)	**1.257 (1.11-1.42)**	**1.130 (1.00-1.28)**

eTable 4 shows results of Cox regression analysis, Hazard Ratios (95% Confidence Intervals), for all-cause mortality comparing higher SBP variability measures in quintiles to the lowest quintile as a reference in Males. Higher quintiles predicted higher mortality. This relationship continued to be significant after adjustment.

Abbreviations. ARV, average real variability; CV, coefficient of variation; ref, reference; SBP, systolic blood pressure; SD, standard deviation; VIM, variability independent of the mean.

© 2021 Dasa O et al. *JAMA Network Open.*

eTable 5. Hazard Ratios and 95% Confidence Intervals for All-Cause Mortality in Females

	**VIM**		**ARV**		**CV**		**SD**	
Quintile	Crude	Adjusted	Crude	Adjusted	Crude	Adjusted	Crude	Adjusted
Q1	1.0 (ref)	1.0 (ref)	1.0 (ref)	1.0 (ref)	1.0 (ref)	1.0 (ref)	1.0 (ref)	1.0 (ref)
Q2	1.027 (0.90-1.17)	1.021 (0.90-1.16)	1.097 (0.96-1.26)	0.980 (0.85-1.12)	0.995 (0.87-1.14)	0.990 (0.87-1.13)	0.948 (0.83-1.09)	0.909 (0.79-1.04)
Q3	1.012 (0.89-1.15)	0.945 (0.83-1.08)	**1.162 (1.01-1.33)**	1.005 (0.88-1.15)	1.030 (0.90-1.18)	0.973 (0.85-1.11)	1.081 (0.95-1.23)	0.989 (0.87-1.13)
Q4	1.097 (0.97-1.25)	1.044 (0.92-1.19)	**1.252 (1.10-1.43)**	1.029 (0.90-1.18)	**1.135 (1.00-1.290)**	**1.038 (0.91-1.18)**	**1.185 (1.04-1.35)**	1.043 (0.92-1.19)
Q5	**1.303 (1.15-1.47)**	**1.269 (1.12-1.43)**	1.757 (1.55-1.99)	**1.330 (1.17-1.51)**	**1.365 (1.21-1.54)**	**1.272 (1.13 -1.44)**	**1.528 (1.35-1.73)**	**1.274 (1.13-1.44)**

eTable 5 shows results of Cox regression analysis, Hazard Ratios (95% Confidence Intervals), for all-cause mortality comparing higher SBP variability measures in quintiles to the lowest quintile as a reference in Females. Higher quintiles predicted higher mortality in Females stronger than in Males. This relationship continued to be significant after adjustment.

Abbreviations. ARV, average real variability; CV, coefficient of variation; ref, reference; SBP, systolic blood pressure; SD, standard deviation; VIM, variability independent of the mean.

© 2021 Dasa O et al. *JAMA Network Open.*

eTable 6. Hazard Ratios and 95% Confidence Intervals for All-Cause Mortality in the Calcium Channel Blocker Arm

	**VIM**		**ARV**		**CV**		**SD**	
Quintile	Crude	Adjusted	Crude	Adjusted	Crude	Adjusted	Crude	Adjusted
Q1	1.0 (ref)	1.0 (ref)	1.0 (ref)	1.0 (ref)	1.0 (ref)	1.0 (ref)	1.0 (ref)	1.0 (ref)
Q2	0.97 (0.85-1.10)	0.96 (0.85-1.09)	1.10 (0.96-1.25)	1.00 (0.87-1.13)	0.95 (0.84-1.08)	0.93 (0.82-1.05)	0.98 (0.86-1.11)	0.94 (0.83-1.07)
Q3	1.04 (0.92-1.18)	0.97 (0.86-1.10)	1.14 (1.00-1.30)	1.00 (0.87-1.13)	1.05 (0.92-1.19)	1.00 (0.87-1.12)	1.06 (0.94-1.21)	0.98 (0.87-1.12)
Q4	**1.13 (1.00-1.28)**	1.09 (0.96-1.23)	**1.36 (1.20-1.55)**	**1.15 (1.01-1.30)**	**1.16 (1.02-1.31)**	1.07 (0.95-1.21)	**1.25 (1.11-1.41)**	1.11 (0.98-1.26)
Q5	**1.22 (1.08-1.38)**	**1.15 (1.02-1.30)**	**1.61 (1.42-1.82)**	**1.23 (1.08-1.40)**	**1.30 (1.15-1.47)**	**1.14 (1.01-1.30)**	**1.42 (1.25-1.60)**	**1.15 (1.01-1.31)**

eTable 6 shows results of Cox regression analysis, Hazard Ratios (95% Confidence Intervals), for all-cause mortality comparing higher SBP variability measures in quintiles to the lowest quintile as a reference in the calcium channel blocker arm from the original INVEST trial. Higher quintiles predicted higher mortality. This relationship continued to be significant after adjustment. Abbreviations. ARV, average real variability; CV, coefficient of variation; ref, reference; SBP, systolic blood pressure; SD, standard deviation; VIM, variability independent of the mean.

© 2021 Dasa O et al. *JAMA Network Open.*

eTable 7. Hazard Ratios and 95% Confidence Intervals for All-Cause Mortality in the Beta-Blocker Arm

	**VIM**		**ARV**		**CV**		**SD**	
Quintile	Crude	Adjusted	Crude	Adjusted	Crude	Adjusted	Crude	Adjusted
Q1	1.0 (ref)	1.0 (ref)	1.0 (ref)	1.0 (ref)	1.0 (ref)	1.0 (ref)	1.0 (ref)	1.0 (ref)
Q2	0.96 (0.85-1.10)	0.98 (0.86-1.12)	0.99 (0.87-1.13)	0.97 (0.85-1.10)	0.95 (0.83-1.08)	0.97 (0.85-1.10)	0.92 (0.81-1.05)	0.93 (0.81-1.10)
Q3	0.97 (0.85-1.10)	0.95 (0.84-1.08)	0.99 (0.87-1.13)	0.91 (0.80-1.04)	1.00 (0.88-1.14)	0.98 (0.86-1.11)	1.01 (0.89-1.15)	0.96 (0.84-1.10)
Q4	1.03 (0.91-1.16)	1.00 (0.88-1.14)	1.09 (0.96-1.24)	0.95 (0.84-1.10)	1.01 (0.89-1.14)	0.97 (0.85-1.10)	1.01 (0.89-1.14)	0.94 (0.83-1.07)
Q5	**1.14 (1.01-1.28)**	**1.14 (1.01-1.30)**	**1.45 (1.30-1.64)**	**1.14 (1.00-1.30)**	**1.21 (1.08-1.40)**	**1.15 (1.02-1.30)**	**1.31 (1.16-1.47)**	**1.12 (0.99-1.27)**

eTable 7 shows results of Cox regression analysis, Hazard Ratios (95% Confidence Intervals), for all-cause mortality comparing higher SBP variability measures in quintiles to the lowest quintile as a reference in the beta-blocker arm from the original INVEST trial. Higher quintiles predicted higher mortality. This relationship continued to be significant after adjustment.
Abbreviations. ARV, average real variability; CV, coefficient of variation; ref, reference; SBP, systolic blood pressure; SD, standard deviation; VIM, variability independent of the mean.

© 2021 Dasa O et al. *JAMA Network Open.*

eTable 8. Pertinent Baseline Patient Characteristics Across Quintiles of VIM

Characteristic*	VIM Quintiles					Total	*P*-value†
	Q1	Q2	Q3	Q4	Q5		
**N**	3072	3073	3073	3073	3072	15363	
**Age, years**	66.16 (9.73)	66.00 (9.83)	66.38 (10.09)	66.77 (9.93)	67.16 (9.85)	66.53 (9.92)	<.0001
**BMI, Kg/m^2**	29.82 (5.69)	28.93 (5.97)	29.45 (5.62)	29.46 (5.74)	28.99 (5.92)	29.53 (5.84)	<.0001
**Female**	1601 (19.38)	1594 (19.29)	1585 (19.18)	1697 (20.54)	1785 (21.6)	8262 (53.78)	<.0001
**Race**							
White	1420 (20.41)	1426 (20.49)	1417 (20.36)	1382 (19.86)	1314 (18.88)	6959 (45.3)	0.015
Black	466 (20.1)	468 (20.19)	482 (20.79)	460 (19.84)	442 (19.07)	2318 (15.09)	
Hispanic	1111 (19.64)	1106 (19.55)	1090 (19.27)	1129 (19.96)	1221 (21.58)	5657 (36.82)	
Other	75 (17.48)	73 (17.02)	84 (19.58)	102 (23.78)	95 (22.14)	429 (2.79)	
**Baseline SBP, mm Hg**	141.34 (15.06)	143.96 (15.53)	147.43 (17.23)	150.47 (18.18)	158.04 (22.75)	148.23 (18.90)	<.0001
**Baseline DBP, mm Hg**	87.63 (12.14)	85.50 (11.05)	84.81 (10.87)	84.08 (10.41)	83.25 (9.87)	85.00 (11.05)	<.0001
**Overall Mean SBP, mm Hg**	139.34 (14.35)	138.97 (13.32)	139.42 (13.45)	139.92 (13.25)	140.18 (13.93)	139.57 (13.67)	0.004

© 2021 Dasa O et al. *JAMA Network Open.*

**Overall Mean DBP, mm Hg**	81.67 (7.40)	80.84 (7.50)	80.60 (7.81)	80.07 (7.93)	78.86 (8.60)	80.41 (7.91)	<.0001
**History of:**							
Diabetes mellitus	893 (19.79)	895 (19.83)	931 (20.63)	911 (20.19)	883 (19.57)	4513 (29.38)	0.70
Hypercholesterolemia	1683 (19.49)	1779 (20.61)	1751 (20.28)	1727 (20)	1693 (19.61)	8633 (56.19)	0.08
Renal insufficiency	50 (16.67)	58 (19.33)	62 (20.67)	60 (20)	70 (23.33)	300 (1.95)	0.47
Prior MI	887 (20.1)	893 (20.23)	872 (19.76)	914 (20.71)	848 (19.21)	4414 (28.73)	0.43
CHF	156 (20.86)	144 (19.25)	155 (20.72)	135 (18.05)	158 (21.12)	748 (4.87)	0.61
Coronary Revascularization (CABG and/or PCI)	909 (20.27)	978 (21.81)	914 (20.38)	870 (19.4)	813 (18.13)	4484 (29.19)	0.0001
Evidence of LVH	471 (19.42)	462 (19.05)	489 (20.16)	495 (20.41)	508 (20.95)	2425 (15.78)	0.50
PAD	325 (16.68)	356 (18.27)	410 (21.04)	429 (22.01)	429 (22.01)	1949 (12.69)	<.0001
TIA or stroke	214 (19.04)	206 (18.33)	224 (19.93)	241 (21.44)	239 (21.26)	1124 (7.32)	0.34

eTable 8 displays baseline characteristics across quintiles of SBP VIM.
* Data are expressed as mean (SD) or n (%) unless otherwise indicated. (%) in rows across quintiles represent row percentages. Frequency missing = 1325.
† *P*-value form Chi-square or ANOVA tests.
1= Lowest (reference) quintile; 5= Highest quintile.
Abbreviations. BMI, body mass index; n, number; CABG, coronary artery bypass surgery; CHF, congestive heart failure; DBP, Diastolic Blood Pressure; LVH, left ventricular hypertrophy; MI, myocardial infarction; PAD, peripheral artery disease; Q, quintile; SBP, Systolic Blood Pressure; TIA, trainset ischemic attack; VIM, variability independent of the mean.

© 2021 Dasa O et al. *JAMA Network Open.*

eTable 9. Pertinent Baseline Patient Characteristics Across Quintiles of ARV

Characteristic*	ARV Quintiles					Total	*P*-value †
	Q1	Q2	Q3	Q4	Q5		
**N**	3035	3120	3066	3073	3069	15363	
**Age, years**	65.86 (9.69)	65.96 (9.84)	65.94 (9.91)	66.86 (10.00)	67.86 (9.88)	66.53 (9.92)	<.0001
**BMI, Kg/m^2**	29.56 (5.60)	29.64 (5.86)	29.74 (5.74)	29.54 (5.89)	29.17 (5.89)	29.53 (5.84)	0.002
**Female**	1563 (18.92)	1646 (19.92)	1624 (19.66)	1624 (19.66)	1805 (21.85)	8262 (53.78)	<.0001
**Race**							
White	1271 (18.26)	1409 (20.25)	1452 (20.87)	1462 (21.01)	1365 (19.61)	6959 (45.30)	<.0001
Black	387 (16.70)	421 (18.16)	460 (19.84)	465 (20.06)	585 (25.24)	2318 (15.09)	
Hispanic	1312 (23.19)	1201 (21.23)	1061 (18.76)	1061 (18.76)	1022 (18.07)	5657 (36.82)	
Other	65 (15.15)	89 (20.75)	93 (21.68)	85 (19.81)	97 (22.61)	429 (2.79)	
**Baseline SBP, mm Hg**	139.42 (13.85)	144.59 (15.70)	148.41 (17.56)	151.63 (19.32)	157.15 (21.89)	148.23 (18.90)	<.0001
**Baseline DBP, mm Hg**	83.11 (9.72)	84.51 (10.18)	85.41 (11.24)	85.57 (11.30)	86.66 (12.07)	85.00 (11.05)	<.0001
**Overall Mean SBP, mm Hg**	134.89 (12.25)	136.63 (11.78)	138.58 (12.77)	141.01 (13.18)	146.72 (15.00)	139.57 (13.67)	<.0001
**Overall Mean DBP, mm Hg**	80.01 (6.98)	79.91 (7.37)	79.72 (7.76)	80.30 (8.04)	82.11 (9.04)	80.41 (7.91)	<.0001
**History of:**							
Diabetes mellitus	846	885	909	907	966	4513	0.02

© 2021 Dasa O et al. *JAMA Network Open.*

	(18.75)	(19.61)	(20.14)	(20.10)	(21.40)	(29.38)	
Hypercholesterolemia	1636 (18.95)	1712 (19.83)	1775 (20.56)	1779 (20.61)	1731 (20.05)	8633 (56.19)	0.003
Renal insufficiency	40 (13.33)	55 (18.33)	60 (20.00)	50 (16.67)	95 (31.67)	300 (1.95)	<.0001
Prior MI	803 (18.19)	889 (20.14)	915 (20.73)	905 (20.50)	902 (20.43)	4414 (28.73)	0.03
CHF	133 (17.78)	149 (19.92)	146 (19.52)	151 (20.19)	169 (22.59)	748 (4.87)	0.36
Coronary Revascularization (CABG and/or PCI)	821 (18.31)	928 (20.70)	940 (20.96)	893 (19.92)	902 (20.12)	4484 (29.19)	0.03
Evidence of LVH	406 (16.74)	464 (19.13)	485 (20.00)	513 (21.15)	557 (22.97)	2425 (15.78)	<.0001
PAD	310 (15.91)	382 (19.60)	398 (20.42)	411 (21.09)	448 (22.99)	1949 (12.69)	<.0001
TIA or stroke	195 (17.35)	201 (17.88)	227 (20.20)	226 (20.11)	275 (24.47)	1124 (7.32)	0.0007

eTable 9 displays baseline characteristics across quintiles of SBP ARV.
* Data are expressed as mean (SD) or n (%) unless otherwise indicated. (%) in rows across quintiles represent row percentages. Frequency missing = 1325.
† *P*-value form Chi-square or ANOVA tests.
1= Lowest (reference) quintile; 5= Highest quintile.
Abbreviations. ARV, absolute real variability. BMI, body mass index; n, number; CABG, coronary artery bypass surgery; CHF, congestive heart failure; DBP, Diastolic Blood Pressure; LVH, left ventricular hypertrophy; MI, myocardial infarction; PAD, peripheral artery disease; Q, quintile; SBP, Systolic Blood Pressure; TIA, trainset ischemic attack.

© 2021 Dasa O et al. *JAMA Network Open.*

eTable 10. Pertinent Baseline Patient Characteristics Across Quintiles of CV

Characteristic*	CV Quintiles					Total	*P*-value †
	Q1	Q2	Q3	Q4	Q5		
**N**	3073	3072	3073	3073	3072	15363	
**Age, years**	66.10 (9.71)	65.95 (9.90)	66.33 (9.92)	66.78 (10.03)	67.32 (9.84)	66.53 (9.92)	<.0001
**BMI, Kg/m^2**	29.75 (5.69)	29.88 (5.94)	29.53 (5.63)	29.46 (5.71)	29.01 (5.98)	29.53 (5.84)	<.0001
**Female**	1579 (19.11)	1609 (19.47)	1585 (19.18)	1698 (20.55)	1791 (21.68)	8262 (53.78)	<.0001
**Race**							
White	1411 (20.28)	1410 (20.26)	1418 (20.38)	1381 (19.84)	1339 (19.24)	6959 (45.30)	0.34
Black	455 (19.63)	462 (19.93)	457 (19.72)	474 (20.45)	470 (20.28)	2318 (15.09)	
Hispanic	1135 (20.06)	1124 (19.87)	1114 (19.69)	1114 (19.69)	1170 (20.68)	5657 (36.82)	
Other	72 (16.78)	76 (17.72)	84 (19.58)	104 (24.24)	93 (21.68)	429 (2.79)	
**Baseline SBP, mm Hg**	140.01 (14.38)	143.18 (15.42)	146.76 (16.31)	151.31 (18.02)	159.98 (22.42)	148.23 (18.90)	<.0001
**Baseline DBP, mm Hg**	82.85 (9.67)	83.89 (10.45)	84.69 (10.72)	85.80 (11.22)	88.07 (12.07)	85.00 (11.05)	<.0001
**Overall Mean SBP, mm Hg**	138.01 (13.65)	138.08 (13.42)	138.71 (12.86)	140.62 (13.40)	142.42 (14.45)	139.57 (13.67)	<.0001
**Overall Mean DBP, mm Hg**	81.55 (7.44)	80.72 (7.48)	80.45 (7.76)	80.19 (8.02)	79.14 (8.62)	80.41 (7.91)	<.0001
**History of:**							
Diabetes mellitus	880	895	927	907	904	4513	0.76

© 2021 Dasa O et al. *JAMA Network Open.*

	(19.50)	(19.83)	(20.54)	(20.10)	(20.03)	(29.38)	
Hypercholesterolemia	1698 (19.67)	1764 (20.43)	1758 (20.36)	1719 (19.91)	1694 (19.62)	8633 (56.19)	0.22
Renal insufficiency	51 (17.00)	52 (17.33)	58 (19.33)	65 (21.67)	74 (24.67)	300 (1.95)	0.18
Prior MI	898 (20.34)	874 (19.80)	872 (19.76)	902 (20.43)	868 (19.66)	4414 (28.73)	0.81
CHF	158 (21.12)	137 (18.32)	157 (20.99)	140 (18.72)	156 (20.86)	748 (4.87)	0.57
Coronary Revascularization (CABG and/or PCI)	914 (20.38)	949 (21.16)	931 (20.76)	877 (19.56)	813 (18.13)	4484 (29.19)	0.001
Evidence of LVH	469 (19.34)	446 (18.39)	489 (20.16)	492 (20.29)	529 (21.81)	2425 (15.78)	0.06
PAD	332 (17.03)	346 (17.03)	418 (17.03)	420 (17.03)	433 (17.03)	1949 (12.69)	<.0001
TIA or stroke	211 (18.77)	202 (17.97)	234 (20.82)	224 (19.93)	253 (22.51)	1124 (7.32)	0.11

eTable 10 displays baseline characteristics across quintiles of SBP CV.
* Data are expressed as mean (SD) or n (%) unless otherwise indicated. (%) in rows across quintiles represent row percentages. Frequency missing = 1325.
† *P*-value form Chi-square or ANOVA tests.
1= Lowest (reference) quintile; 5= Highest quintile.
Abbreviations. BMI, body mass index; n, number; CABG, coronary artery bypass surgery; CHF, congestive heart failure; CV, coefficient of variation; DBP, Diastolic Blood Pressure; LVH, left ventricular hypertrophy; MI, myocardial infarction; PAD, Q, quintile; peripheral artery disease; SBP, Systolic Blood Pressure; TIA, trainset ischemic attack.

© 2021 Dasa O et al. *JAMA Network Open.*

eTable 11. Pertinent Baseline Patient Characteristics Across Quintiles of SD

Characteristic*	SD Quintiles					Total	*P*-value †
	Q1	Q2	Q3	Q4	Q5		
**N**	3074	3071	3073	3073	3072	15363	
**Age, years**	66.04 (9.73)	65.57 (9.79)	66.36 (9.99)	66.76 (9.94)	67.75 (9.88)	66.53 (9.92)	<.0001
**BMI, Kg/m^2**	29.63 (5.66)	29.91 (5.79)	29.48 (5.77)	29.56 (5.81)	29.05 (5.92)	29.53 (5.84)	<.0001
**Female**	1561 (18.89)	1606 (19.44)	1605 (19.43)	1668 (20.19)	1822 (22.05)	8262 (53.78)	<.0001
**Race**							
White	1382 (19.86)	1416 (20.35)	1432 (20.58)	1374 (19.74)	1355 (19.47)	6959 (45.30)	0.0007
Black	416 (17.95)	464 (20.02)	423 (18.25)	503 (21.70)	512 (22.09)	2318 (15.09)	
Hispanic	1210 (21.39)	1107 (19.57)	1131 (19.99)	1101 (19.46)	1108 (19.59)	5657 (36.82)	
Other	66 (15.38)	84 (19.58)	87 (20.28)	95 (22.14)	97 (22.61)	429 (2.79)	
**Baseline SBP, mm Hg**	137.67 (13.62)	141.54 (14.27)	146.64 (15.15)	152.26 (17.07)	163.15 (21.74)	148.23 (18.90)	<.0001
**Baseline DBP, mm Hg**	82.8 (9.53)	83.43 (10.23)	84.77 (10.42)	86.31 (11.40)	88.59 (12.06)	85.00 (11.05)	<.0001
**Overall Mean SBP, mm Hg**	135.56 (12.93)	136.26 (12.29)	138.13 (12.23)	141.18 (12.79)	146.72 (14.86)	139.57 (13.67)	<.0001
**Overall Mean DBP, mm Hg**	80.11 (7.41)	79.46 (7.48)	79.70 (7.67)	80.64 (8.00)	82.12 (8.66)	80.41 (7.91)	<.0001
**History of:**							
Diabetes mellitus	867	901	900	919	926	4513	0.50

© 2021 Dasa O et al. *JAMA Network Open.*

	(19.21)	(19.96)	(19.94)	(20.36)	(20.52)	(29.38)	
Hypercholesterolemia	1692 (19.60)	1786 (20.69)	1736 (20.11)	1737 (20.12)	1682 (19.48)	8633 (56.19)	0.05
Renal insufficiency	41 (13.67)	62 (20.67)	53 (17.67)	62 (20.67)	82 (27.33)	300 (1.95)	0.004
Prior MI	888 (20.12)	898 (20.34)	860 (19.48)	874 (19.80)	894 (20.25)	4414 (28.73)	0.81
CHF	154 (20.59)	150 (20.05)	148 (19.79)	146 (19.52)	150 (20.05)	748 (4.87)	0.99
Coronary Revascularization (CABG and/or PCI)	899 (20.05)	980 (21.86)	904 (20.16)	872 (19.45)	829 (18.49)	4484 (29.19)	0.0007
Evidence of LVH	464 (19.13)	435 (17.94)	488 (20.12)	493 (20.33)	545 (22.47)	2425 (15.78)	0.003
PAD	331 (16.98)	362 (18.57)	420 (21.55)	405 (20.78)	431 (22.11)	1949 (12.69)	0.0003
TIA or stroke	207 (18.42)	206 (18.33)	227 (20.20)	213 (18.95)	271 (24.11)	1124 (7.32)	0.007

eTable 11 displays baseline characteristics across quintiles of SBP SD.
* Data are expressed as mean (SD) or n (%) unless otherwise indicated. (%) in rows across quintiles represent row percentages. Frequency missing = 1325.
† *P*-value form Chi-square or ANOVA tests.
1= Lowest (reference) quintile; 5= Highest quintile.
Abbreviations. BMI, body mass index; n, number; CABG, coronary artery bypass surgery; CHF, congestive heart failure; DBP, Diastolic Blood Pressure; LVH, left ventricular hypertrophy; MI, myocardial infarction; PAD, peripheral artery disease; Q, quintile; SBP, Systolic Blood Pressure; SD, standard deviation; TIA, trainset ischemic attack.

© 2021 Dasa O et al. *JAMA Network Open.*

eTable 12. Cochran-Armitage Test for Trend Across Blood Pressure Variability Quintiles

	P for trend*	
	SBP	DBP
VIM quintiles	<.0001	<.0001
ARV quintiles	<.0001	<.0001
CV quintiles	<.0001	<.0001
SD quintiles	<.0001	0.074

*2-sided *P*-value from Cochran-Armitage test for trend testing a binary outcome variable (death).

Abbreviations. ARV, average real variability; CV, coefficient of variation; DBP, diastolic blood pressure; SBP, systolic blood pressure; SD, standard deviation; VIM, variability independent of the mean.

© 2021 Dasa O et al. *JAMA Network Open.*

eBox. Blood Pressure Variability Equations

$$ARV = \frac{1}{n-1}\sum_{i=1}^{n-1}\left|BP_{i+1} - BP_i\right|$$

$$VIM = \frac{SD}{\overline{\overline{BP^x}}}\ x\ \overline{BP_{Pop}^x}$$

$$CV = \frac{SD}{\overline{\overline{BP}}}$$

$$SD = \sqrt{\frac{\sum(\boldsymbol{BP}_i - \overline{BP})^2}{n-1}}$$

Abbreviations. ARV, average real variability; BP, blood pressure; CV, coefficient of variation; SD, standard deviation; VIM, variability independent of the mean.
BP pop = Population mean blood pressure.
i = represent the ith BP measurement or the Blood Pressure at the ith visit.
x = the power imputed from non-linear regression

© 2021 Dasa O et al. *JAMA Network Open.*

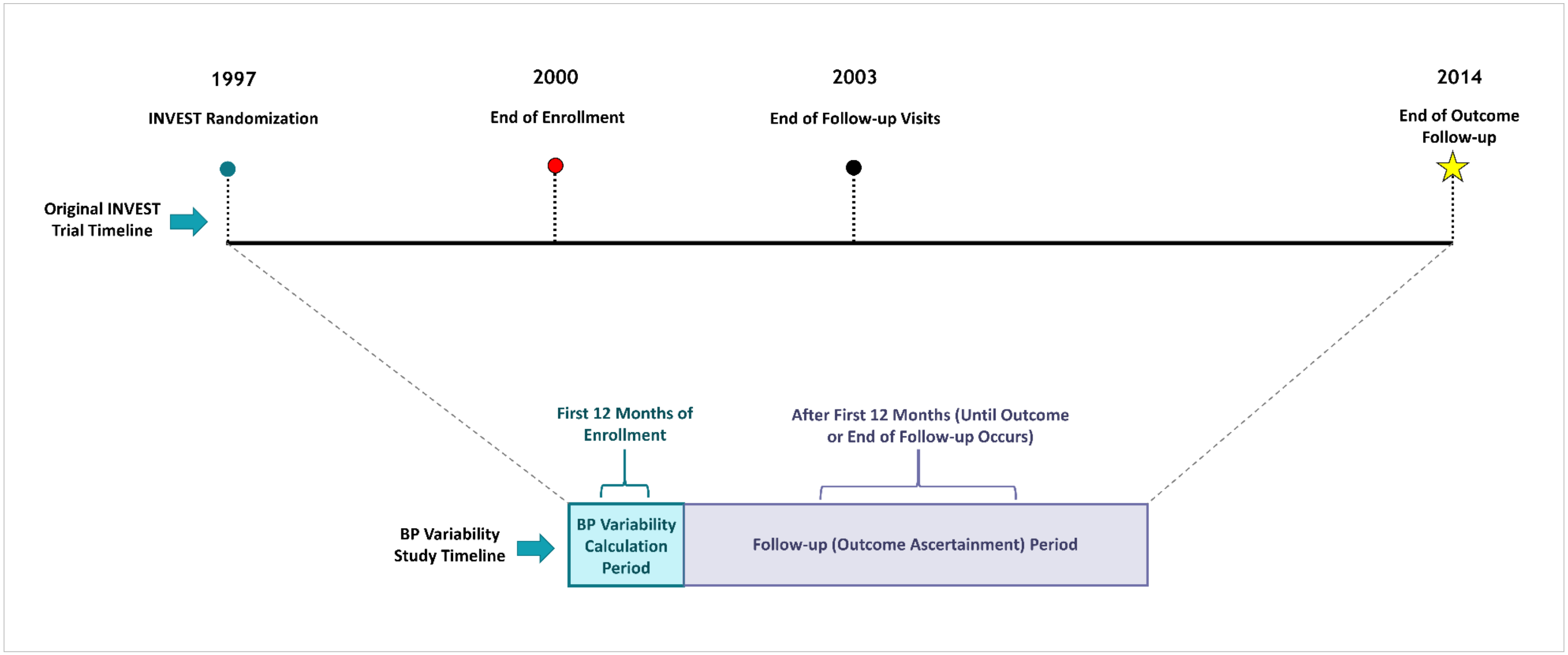

eFigure 1. Study Timeline

The upper line illustrates the Original INVEST study timeline from randomization to end of follow-up visits. In 2014, a National Death Index query was requested. The lower line illustrates the current study timeline including blood pressure ascertainment period (1st year of enrollment).

Abbreviations. BP, blood pressure; INVEST, INternational VErapamil SR-Trandolapril Study.

© 2021 Dasa O et al. *JAMA Network Open.*

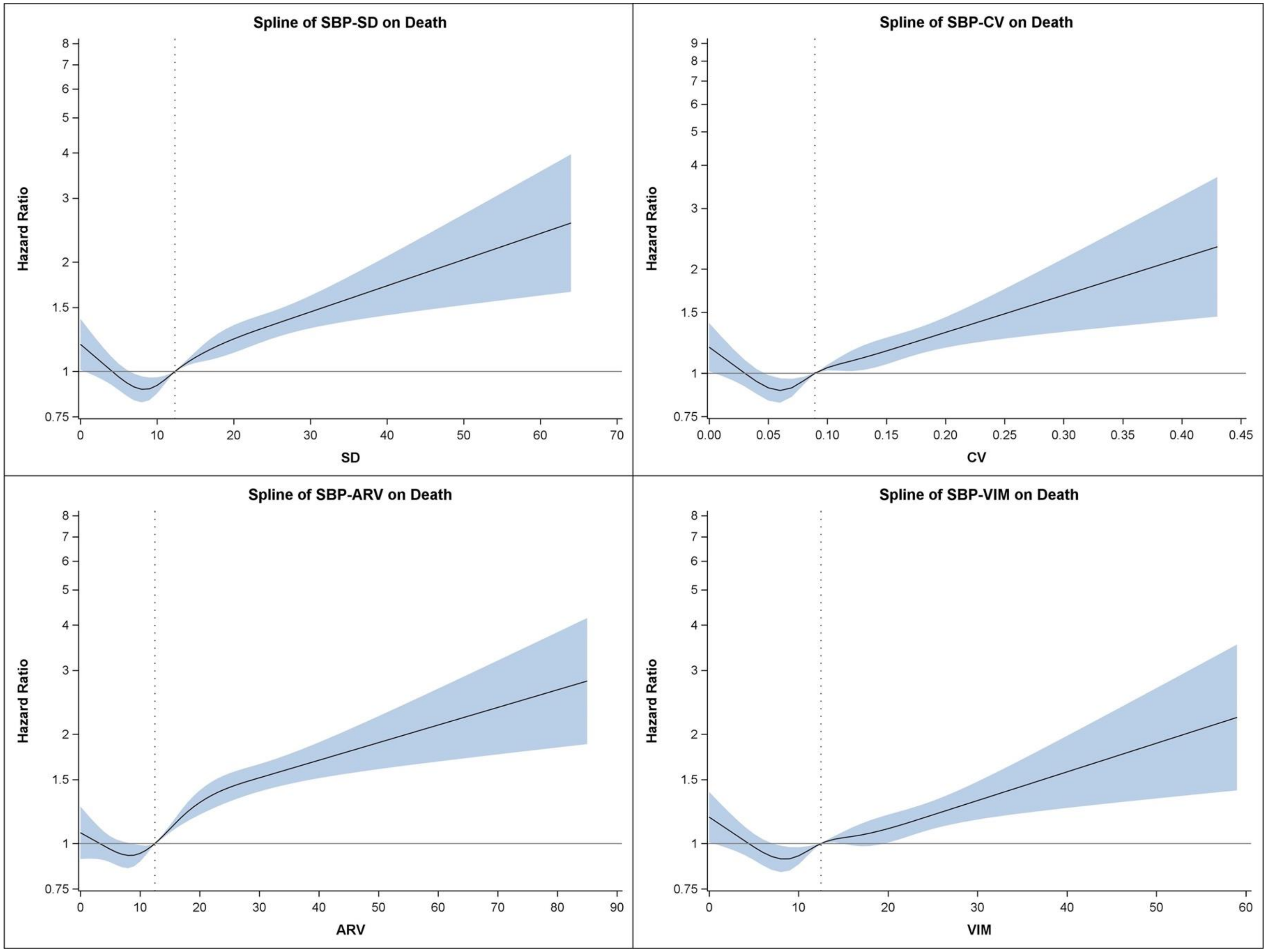

eFigure 2. Spline Curves of Systolic Blood Pressure Variability Measures on Primary Outcome

© 2021 Dasa O et al. *JAMA Network Open.*

Spline curves demonstrating the association between each systolic blood pressure variability measures modeled as a continuous variable with the primary outcome (unadjusted), using Cox proportional hazard models and restricted quadratic splines with knots at 5th, 27.5th, 50th, 72.5th, 95th percentiles of each BP variability measure.
Abbreviations. ARV, average real variability; SBP, systolic blood pressure; CV, coefficient of variation; SD, standard deviation; VIM, variability independent of the mean.

© 2021 Dasa O et al. *JAMA Network Open.*

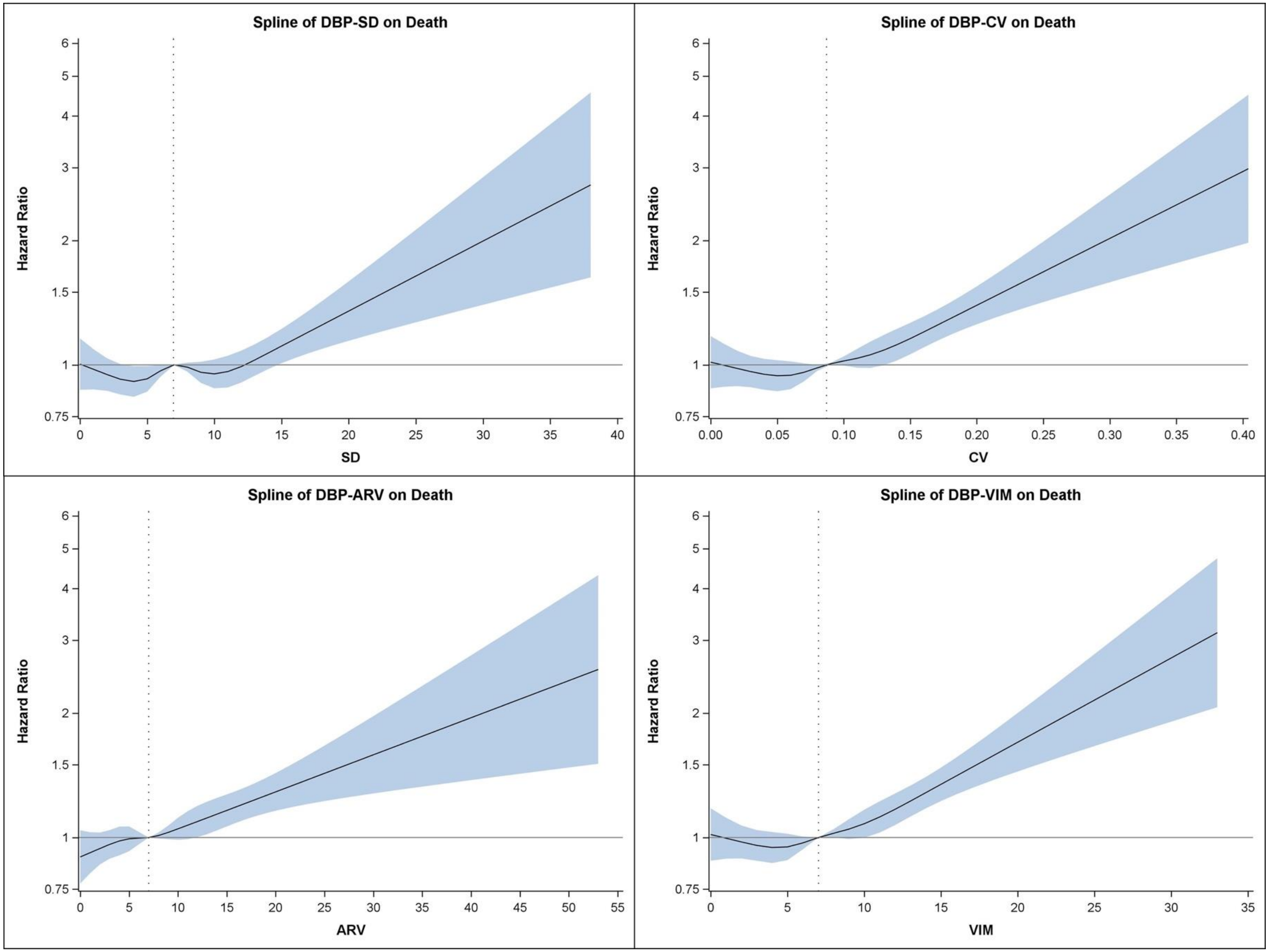

eFigure 3. Spline Curves of Diastolic Blood Pressure Variability Measures on Primary Outcome

© 2021 Dasa O et al. *JAMA Network Open.*

Spline curves demonstrating the association between each diastolic blood pressure variability measures as a continuous variable with the primary outcome (unadjusted) using Cox proportional hazard models and restricted quadratic splines with knots at 5th, 27.5th, 50th, 72.5th, 95th percentiles of each BP variability measure.
Abbreviations. ARV, average real variability; SBP, systolic blood pressure; CV, coefficient of variation; SD, standard deviation; VIM, variability independent of the mean.

© 2021 Dasa O et al. *JAMA Network Open.*

eFigure 4. Schematic Diagram of Implementing BP Variability Calculation in Clinical Practice

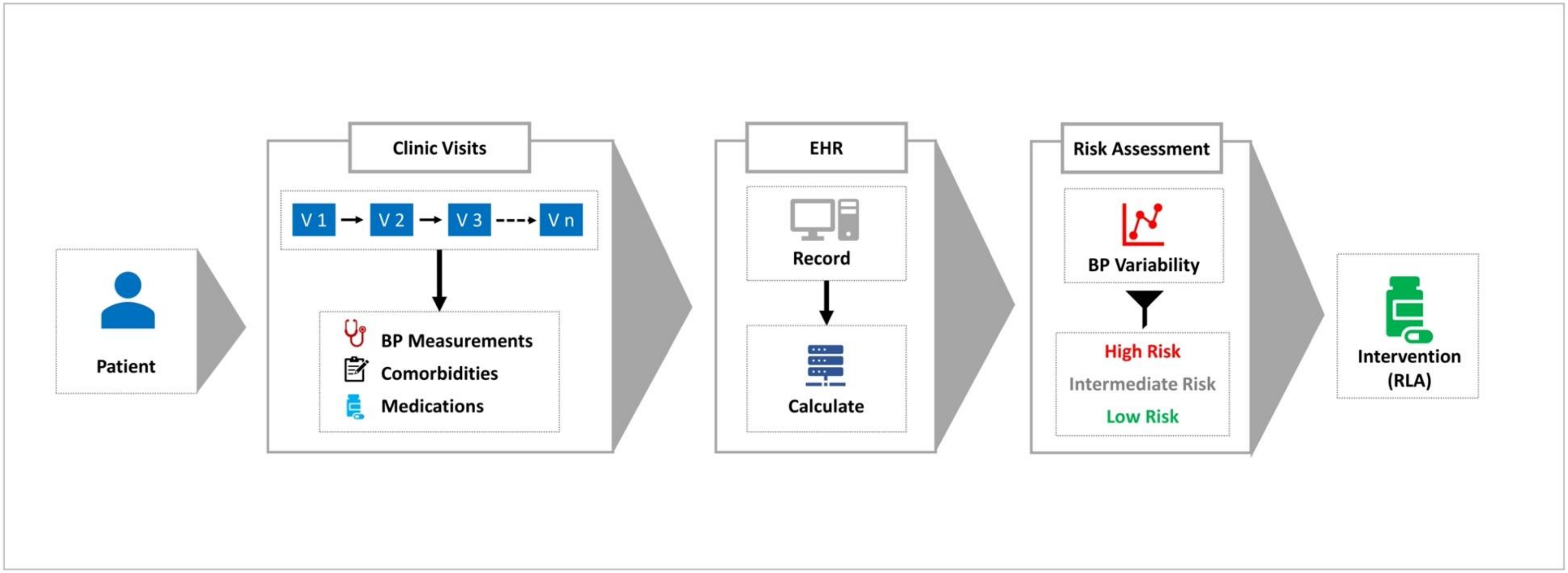

With electronic medical records, blood pressures over several visits can easily be analyzed for multiple blood pressure variability measures. Using pertinent patients' demographics and possibly medications, predictions models can developed be evaluate the excess risk and long-term outcomes that are related to higher blood pressure variability. Once risk is assessed, patients can be divided into three risk groups: "high", "intermediate" and "low" for risk lowering approach (RLA). High risk patients would require "greatly accelerated" RLA, i.e., intensive medical management. Moderate risk patients require "modest acceleration" in RLA, while low risk patients can maintain usual care but with continued follow-up to periodically reassess risk. Gaps in knowledge still exist on what interventions should be triggered when high variability is detected (i.e., change in medications, increased surveillance, more aggressive primary prevention measures…etc.).
Abbreviations. BP, blood pressure; EHR, electronic health record; RLA, risk lowering approach; V, visit.

© 2021 Dasa O et al. *JAMA Network Open.*
